# A comparison of the metabolic effects of sustained strenuous activity in polar environments on men and women

**DOI:** 10.1038/s41598-020-70296-4

**Published:** 2020-08-17

**Authors:** John Hattersley, Adrian J. Wilson, Rob Gifford, Jamie Facer-Childs, Oliver Stoten, Rinn Cobb, C. Doug Thake, Rebecca M. Reynolds, David Woods, Chris Imray

**Affiliations:** 1 Coventry NIHR CRF Human Metabolic Research Unit, University Hospitals Coventry and Warwickshire NHS Trust, Coventry, CV2 2DX UK; 2 School of Engineering, University of Warwick, Coventry, CV4 7AL USA; 3 Faculty of Health and Life Sciences, Coventry University, Coventry, UK; 4 Department of Physics, University of Warwick, Coventry, CV4 7AL UK; 5 University/British Heart Foundation Centre for Cardiovascular Science, Queen’s Medical Research Institute, University of Edinburgh, Edinburgh, UK; 6 Research and Clinical Innovation, Royal Centre for Defence Medicine, Birmingham, UK; 7 Institute of Child Health, University College London, London, WC1N 1EH UK; 8 Emergency Department, Royal Bournemouth Hospital, Bournemouth, BH7 7DW UK; 9 Performance, Nutrition and Dietetic Consulting, pnd consulting.co.uk, Middlesbrough, UK; 10 Research Institute for Sport, Physical Activity and Leisure, Leeds Beckett University, Leeds, UK; 11 Northumbria and Newcastle NHS Trusts, Wansbeck General and Royal Victoria Infirmary, Newcastle, UK; 12 Department of Vascular and Renal Transplant Surgery, University Hospitals Coventry and Warwickshire NHS Trust, Coventry, CV2 2DX UK

**Keywords:** Endocrinology, Medical research

## Abstract

This study investigates differences in pre- to post-expedition energy expenditure, substrate utilisation and body composition, between the all-male Spear17 (SP-17) and all-female Ice Maiden (IM) transantarctic expeditions (IM: N = 6, 61 days, 1700 km; SP-17: N = 5, 67 days, 1750 km). Energy expenditure and substrate utilisation were measured by a standardised 36 h calorimetry protocol; body composition was determined using air displacement plethysmography. Energy balance calculation were used to assess the physical challenge. There was difference in the daily energy expenditure (IM: 4,939 kcal day^−1^; SP-17: 6,461 kcal day^−1^, *p* = 0.004); differences related to physical activity were small, but statistically significant (IM = 2,282 kcal day^−1^; SP-17 = 3,174 kcal day^−1^; *p* = 0.004). Bodyweight loss was modest (IM = 7.8%, SP-17 = 6.5%; *p* > 0.05) as was fat loss (IM = 30.4%, SP-17 = 40.4%; *p* > 0.05). Lean tissue weight change was statistically significant (IM = − 2.5%, SP-17 = + 1.0%; *p* = 0.05). No difference was found in resting or sleeping energy expenditure, normalised to lean tissue weight (*p* > 0.05); nor in energy expenditure when exercising at 80, 100 and 120 steps min^−1^, normalised to body weight (*p* > 0.05). Similarly, no difference was found in the change in normalised substrate utilisation for any of the activities (*p* > 0.05). Analysis suggested that higher daily energy expenditures for the men in Spear-17 was the result of higher physical demands resulting in a reduced demand for energy to thermoregulate compared to the women in Ice Maiden. The lack of differences between men and women in the change in energy expenditure and substrate utilisation, suggests no sex difference in response to exposure to extreme environments.

## Introduction

There is an increasing involvement of women in extreme activities often in adverse environmental conditions that are characterised by a deficit in Energy Availability (EA), the difference between the calorific intake and the energy expended. These activities, which include extreme sports^1^, expeditionary travel and military combat training^2^, have traditionally been undertaken by men with the result that the majority of the research looking at physiological adaptation and responses, particularly during expeditionary travel in polar regions and to altitude, has been on men. Therefore, there is a paucity of knowledge regarding the similarity and differences in the adaption of women to high levels of physical activity in extreme environments.

Antarctica is the coldest and highest continent on earth with summer temperatures down to − 70 °C and an average elevation above the sea of 2,400 m (maximum elevation is Mount Vinson at 4,892 m) and therefore presents multiple challenges to those exploring it. Whilst the elevations in Antarctica are modest when considered against the Himalayan or Karakoram peaks, some studies^3,4^ but not all^5^ have shown a prioritised utilisation of glucose over fatty acids in male subjects exposed to the altitudes encountered in Antarctica that were not found in female subjects exposed to similar altitudes^6^. The primary impact of the low temperatures in Antarctica is to increase the basal metabolic rate (BMR) to maintain core temperature. A non-expedition study containing both male and female participants which modelled the components of the metabolic rate showed that the increase in BMR required for thermoregulation in high ambient temperatures was less than that required in low temperatures^7^. Unfortunately, these data were not partitioned on sex and therefore it is not possible to determine whether the changes were the same for male and female participants. Isotope techniques can be used to measure time-averaged non-protein metabolism^8^ and time-averaged protein metabolism^9^ during expeditions^10–13^. From earlier expeditionary studies the measured energy expenditure was found to be much higher than that predicted from activity scores^14^ and a 60% increase was found in the BMR^15^ which was attributed to increased thyroid activity. This increase in BMR is consistent with small levels of weight loss seen in men undertaking low levels of physical work whilst over-wintering in Antarctica^16^. In a recent analysis of energy expenditure and substrate utilisation on an all-male transantarctic expedition (Spear-17), we found that this 60% increase in BMR was consistent with an increase in the energy required for thermoregulation^17^. The same analysis on data from participants in an all-female transantarctic expedition of similar length and duration (Ice Maiden) did not give a similar difference in BMR^18^.

Participants in supported polar expeditions have traditionally experienced modest levels of negative energy availability^19–22^ whilst those undertaking unsupported polar expeditions have experienced high levels of negative energy availability as evidenced by up to 25% loss of body weight^10,12^. Our previous work has suggested only modest levels of negative energy availability in participants in both the Spear-17^17^ and Ice Maiden^18^ expeditions. Negative energy availability has been postulated as a possible cause of the hypothalamic pituitary gonad (HPG) axis suppression (termed the female triad) in female participants in extreme physical activities, including military combat training^23^. Whilst women appear to have a higher susceptibility to HPG suppression during extreme physical activity^24^, HPG suppression is also seen in men^1^. However, the impact of HPG axis suppression on energy expenditure is unknown.

It has previously been suggested^3,4,6,25^ that there are potential differences in the way male and female participants respond to extreme physical and physiological demands. We have recently had the opportunity to study energy expenditure and substrate utilisation pre- and post-expedition on participant from two independent polar expeditions (one all male—Spear-17; one all-female—Ice Maiden):

- *Spear-17*—during the Antarctic summer of 2016/17 a team of six male British Army Reservists, including an experienced polar traveller who was their leader, undertook an unassisted crossing of Antarctica on ski (https://www.forces.net/news/feature/spear-17-team-complete-mammoth-antarctic-expedition). The 67 day, 1,750 km journey took them from the Hercules Inlet (altitude 244 m) to the South Pole (altitude 2840 m), where the single resupply took place, then down the Shackleton Glacier, finally finishing on the Ross Sea Ice (altitude < 50 m). During the journey, they experienced temperatures as low as – 57 C whilst pulling sledges weighing up to 120 kg to an altitude of 3350 m. Expedition participants were provided with individually pre-packaged daily food packs which had a mean nutritional value of 27.2 MJ day^−1^ (6,500 kcal day^−1^) with a macronutrient breakdown of 35% carbohydrate, 55% fat and 8% protein^17^. The estimated average consumption was 92%^18^. Neither the calorific value of the diet, nor its composition was not part of the study but were determined by the expeditionary team.
- *Ice Maiden*—during the Antarctic summer of 2017/2018, six women participated in a 1700 km unassisted ski traverse of the Antarctic continent from Leverett Glacier (altitude 244 m) along the McMurdo Route to the South Pole (altitude 2840 m), where the first of two resupplies took place, to Hercules Inlet (altitude 767 m) via the Thiel Mountains, where the second resupply occurred. The team was hauling sledges weighing up to 80 kg. The expedition lasted 61 days with two resupplies. This was the first all-female team to use muscle power alone to ski coast-to-coast across (https://exicemaiden.com). The six participants in this British Army expedition were selected from 250 volunteers, all serving members of the British armed forces, following 2 years of selection and training activities^26^. During the expedition, participants were provided with individually pre-packaged daily food packs which had a mean nutritional value of 20.9 MJ day^−1^ (5,000 kcal day^−1^) with a macronutrient breakdown of 45% carbohydrate, 45% fat and 10% protein with an estimated average consumption of 85%. Neither the caloric value nor the composition of the diet formed part of the study on energy expenditure and substrate utilisation, but were determined by one of the authors (RC) in collaboration with the expeditionary team.

We have previously reported on the energy expenditure and substrate utilisation on participants from each expedition^17,18^. These studies were carried out in a whole-body calorimeter to a standard protocol and present a rare opportunity to gain an insight into differences in the physiological response of men and women undertaking expeditionary polar travel. To allow for changes in body weight and body composition between men and women, in this paper we compare: the fractional change in body composition; the normalised change in energy expenditure and normalised substrate utilisation measured pre- and post-expedition between participants in the two expeditions.

## Materials and methods

### Study participants

There were six male participants in the Spear-17 expedition (age 26–40 years) and six female participants (age 28–36 years) in the Ice Maiden expedition. Participants were invited to participate in a scientific study of the impact of the expedition on their metabolism. For both expeditions, participation in the scientific study was voluntary and independent of their participation in the expedition itself. All six male participants in the Spear-17 expedition and all six female participants in the Ice Maiden expedition volunteered to be participants in the scientific studies and gave written consent prior to any data collection. Only five members of Spear-17 completed the expedition, therefore data from the sixth participant was excluded from the analysis presented in this paper. Ethics approval for the Spear-17 study was obtained from the National Health Authority Research Ethics committee, West Midlands—Solihull (ID: 13/WM/0327), Ministry of Defence—Research Ethics Committee and University Hospitals Coventry and Warwickshire Research and Development Governance Committee, under the GAFREC framework (REF: GF0121). Ethics approval for the Ice Maiden study was obtained from the Ministry of Defence Research Ethics Committee (827MoDREC/17). Both studies were carried out in compliance with the Ethical Principles for Medical Research on Human Subjects set down in the Declaration of Helsinki by the World Medical Association. All study protocols and objectives were fully explained to all participants before securing informed written consent.

### Measurements

A common protocol was used to study the energy expenditure of participants from each of the two expeditions on two separate occasions: the first in the two weeks before departure from the UK (2 weeks prior to the start of the expedition) and the second within two weeks following their return to the UK (2 weeks after the expedition successfully concluded).

Full details of the protocol have been published previously^17^ but will be summarised here for completeness. Each participant spent 36 h in a dual whole-body calorimeters (Maastricht Instruments, Netherlands^27,28^). The measurement period incorporated an hour supine rest to measure the resting metabolic rate (RMR), four meals, three exercise sessions and two sleeping periods. Calorimeters allow accurate measurements of oxygen and carbon dioxide, and with the collection of three consecutive 12-h urine samples for urinary protein estimates^29^. The energy expenditure and substrate utilisation were calculated on a minute resolution using standard formulae^30–32^. During the period in the calorimeters the food intake of the participants was isocaloric based on lean tissue mass with a composition typical of a western diet (50% carbohydrate, 35% fat and 15% protein).

To maintain thermal neutrality, the environment within the chamber was controlled at a relative humidity of 57 ± 5% at a temperature of 24 ± 0.5 °C during the day and 22 ± 0.5 °C during the night.

Before arriving for the start of the measurement session at around 6 pm, participants were asked to refrain from eating and drinking tea or coffee from lunchtime onwards. On arrival, the participants had their height and weight measured (Seca 799, Seca, UK) and then their body composition determined by Air Displacement Plethysmography (ADP) using a BodPod 2000A (Cosmed Inc., USA).

### Data analysis

**Figure 1:**
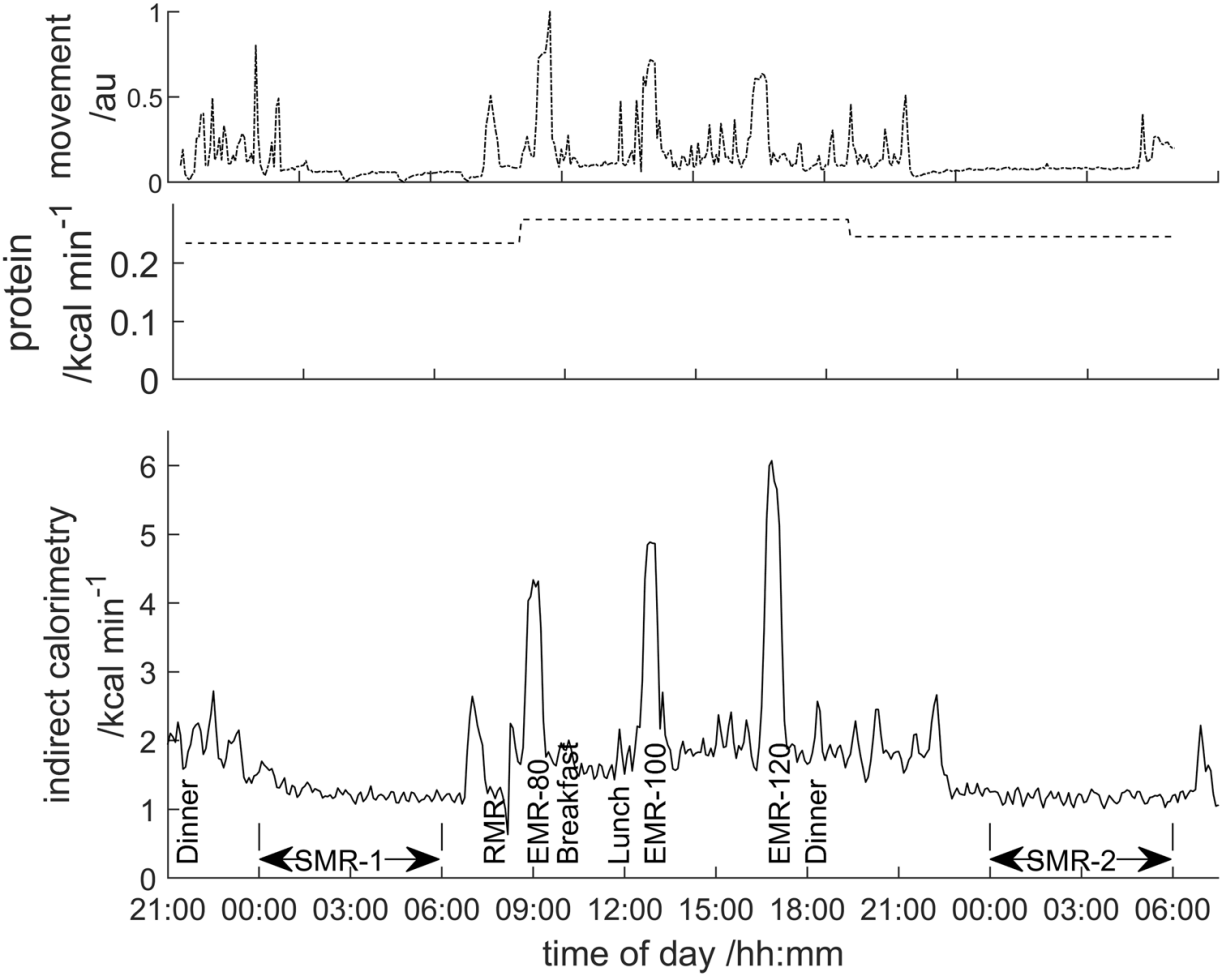
The raw data obtained from the whole body calorimeter studies annotated with the events analysed in this paper and the times when the participants were fed. The (non-protein) energy expenditure calculated from the difference in the concentrations of O_2_ and CO_2_ entering and leaving the room is shown in the lower pane, the protein energy expenditure from the 12-h urine sample analysis in the middle pane and the subject activity from the ultrasound movement sensors, scaled 0–1 is shown in the upper pane.

The metabolic rate was determined on a minute-by-minute basis from the continuous measurements of the difference in concentration of O_2_ and CO_2_ in the gases entering and leaving the room. This, together with the data from the movement sensors, gave a profile of the energy expenditure and activity for the participant over the study period as shown in Fig. 1.

Figure 1 also shows the energy due to protein metabolism where expenditure was estimated from the analysed creatinine and urea in the 12-h urine samples^29^. The total energy expenditure was the sum of the energy expenditure from the O_2_/CO_2_ data and the protein energy expenditure from the urine nitrogen analysis.

In this study we aim to determine differences in the pre- to post-expedition measurements of body composition, energy expenditure and substrate utilisation to determine whether there are any differences between the participants in the all-male (Spear-17) and all-female (Ice Maiden) expeditions. The two expeditions were not identical and therefore to gain an insight into the challenge the expeditions presented, a method of calculating the energy expenditure during the expedition is also described.

### Change in body composition

The change in total body, lean tissue and body fat weight were determined from the pre- and post-expedition measurements. These were expressed as a percentage of pre-expedition values in order to make comparisons between participants in the Spear-17 and Ice Maiden expeditions.

### Change in sleeping metabolic rate (ΔSMR-1, ΔSMR-2)

The metabolic rate during sleep (calculated between 00:00 and 06:00) was determined by the amount of metabolically active tissue in the body. Therefore, energy expenditure and substrate utilisation values were divided by lean tissue weight to give normalised measures and the difference between these pre- and post-expedition for the two sleep periods were used to compare participants from the Spear-17 and Ice Maiden expeditions.

### Change in resting metabolic rate (ΔRMR)

The resting metabolic rate was measured between 07:00 and 08:00 after waking at 06:30 following the first sleep period. The subject was asked to lie quietly on the bed undertaking no activity and not going to sleep. The RMR was calculated as the average metabolic rate for the period 07:20–07:50 when participants were least restless. As with the sleeping metabolic rate, the values would be expected to vary with the weight of lean tissue and so the difference between pre- and post-expedition energy expenditure values normalised to lean tissue weight were used to compare participants from the Spear-17 and Ice-Maiden expeditions.

### Change in metabolic rate during exercise (ΔEMR-80, ΔEMR-100, ΔEMR-120)

Three 30 min periods of stepping exercise were performed using a standard height exercise step (Reebok Aerobic Step, height 150 mm, Reebok, UK) at step rates of 80, 100 and 120 steps min^−1^, with the step rate controlled by a simple metronome (Tempo Perfect, https://www.nch.com.au/metronome). The exercising metabolic rate was calculated as the mean energy expenditure throughout the 30-min exercise period and denoted EMR80, EMR100 and EMR120 for the three different step rates. The stepping exercise is a cyclical change in potential energy and the energy expenditure will depend on the total weight of the subject. Therefore the change in values normalised to the total body weight from the pre- and post-expedition measurements, denoted ΔEMR-80, ΔEMR-100 and ΔEMR-120, were used to compare participants in the Spear-17 and Ice Maiden expeditions.

### Energy expenditure during the expedition

Ignoring diet induced thermogenesis (DIT) which is small^33^, the energy expenditure for a short period of time (e.g. the minute-by-minute values shown in Fig. 1) can be thought of as the sum of the energy to deliver physical activity, the energy for non-shivering thermogenesis and the energy to maintain body functions, the basal metabolic rate (BMR). A 60% increase in BMR during exposure to the Antarctic environment had previously been reported^15^. Assuming this fractional increase in the BMR is the additional energy required to maintain organ function and achieve thermoregulation at rest in a polar region, then the daily energy expenditure due to activity, *E*_*act*_, during the expedition can be estimated from:

1 $$ E_{act} = E_{tot} - BMR_{temp} (1 + T_{f} ) $$

where *E*_*tot*_ is the total daily energy expenditure during the expedition, *BMR*_*temp*_ is the BMR measured in a temperate environment and *T*_*f*_ is the time-averaged fractional increase in the BMR as a result of exposure to the polar environment measured over 24 h. Values of *BMR*_*temp*_ were based on the values of RMR measured at 24 °C during the whole-body calorimeter studies on all participants both pre- and post-expedition. To obtain a value of *E*_*tot*_ the conservation of energy is applied to the energy obtained from the food ingested and the loss of body weight. Assuming a linear loss of lean and fat weight during the expedition and 100% absorption of the food consumed, *E*_*tot*_, in Eq. (1) can be estimated from:

2 $$ E_{tot} = f_{c} E_{nutr} + \frac{{{\Delta }_{fat} ED_{fat} }}{{Ex_{dur} }} + \frac{{{\Delta }_{lean} ED_{lean} }}{{Ex_{dur} }} $$

where *E*_*nutr*_ is the energy available from the supplied nutrition, *f*_*c*_ is the fraction of the supplied nutrition consumed, Δ_*fat*_ is the change in the weight of body fat determined from body composition measurements before and after the expedition, *ED*_*fat*_ is the metabolisable energy density of human body fat, Δ_*lean*_ is the change in the weight of lean body tissue determined from body composition measurements before and after the expedition, *ED*_*lean*_ is the metabolisable energy density of human lean tissue and *Ex*_*dur*_ is the duration of the expedition in days. Using Eqs. (1) and (2) the total daily energy expenditure, *E*_*tot*_, and the daily energy expenditure due to activity, *E*_*act*_, were compared for participants in the two expeditions.

### Statistical analysis

As the number of participants was small it was not possible to robustly determine the normality of the two sets of data. Therefore summarised values for the group of participants are presented as median, lower (Q1) and upper quartile (Q3) of the values. Statistical comparisons between participants in the Spear-17 and Ice Maiden data have used the non-parametric Mann–Whitney U-test for very small samples^34^ with statistical significance taken as *p* ≤ 0.05. We have also analysed the changes for individual participants and how these varied across the two expedition groups.

### Ethics approval

Ethics approval for the Spear-17 study was obtained from the National Health Authority Research Ethics committee, West Midlands—Solihull (ID: 13/WM/0327), Ministry of Defence—Research Ethics Committee and University Hospitals Coventry and Warwickshire Research and Development Governance Committee, under the GAFREC framework (REF: GF0121). Ethics approval for the Ice Maiden study was obtained from the Ministry of Defence Research Ethics Committee (827MoDREC/17). Both studies were carried out in compliance with the Ethical Principles for Medical Research on Human Subjects set down in the Declaration of Helsinki by the World Medical Association. Informed consent was obtained for each participant, for pre and post-expedition measurements.

## Results

For the Spear-17 expedition, five of the six reservists, including their leader, completed the traverse of Antarctica from the Hercules Inlet to the Ross Sea Ice. The sixth reservist left the expedition at the South Pole due to extreme fatigue, and thus their data was excluded from the analysis. All six participants in the Ice Maiden expedition completed the Antarctic crossing from the Leverett Glacier to the Hercules Inlet.

The results for pre- to post-expedition differences energy expenditure and substrate utilisation during sleeping, resting and exercise, and the determination of the energy expenditure during the expedition are given in the following sections. The relationship between these different measures will form part of the Discussion.

### Change in body composition

The median and quartile (Q1; Q3) values for the pre- to post-expedition difference in composition for the Spear-17 expedition were: total body weight [− 5.6 kg (− 7.6 kg; − 4.6 kg)], lean weight [+ 1.0 kg (0.4 kg; 1.1 kg)] and fat weight [− 6.5 kg (− 8.3 kg; − 2.4 kg)]; For the Ice Maiden expedition: total body weight [− 5.8 kg (− 6.7 kg; − 5.0 kg)], lean weight [− 1.9 kg (− 2.0 kg; − 0.9 kg)] and fat tissue weight [− 4.3 kg (− 5.3 kg; − 3.5 kg)]. The median and quartile values for the pre- to post-expedition differences in body composition expressed as a percentage of the pre-expedition values are given in the upper section of Table 1. The changes in body composition were similar between the two expeditions; the only statistically significant difference being between the small gain in lean tissue weight for Spear-17 participants and the loss of lean tissue weight by Ice Maiden participants (*p* < 0.05).

**Table 1:** The median, lower and upper quartiles (Q1; Q3) for the changes between pre- and post-expedition measurements in: body composition, energy expenditure during sleep (ΔSMR-1 and ΔSMR-2) and rest (ΔRMR); and during exercise at 80 steps min^−1^ (ΔEMR-80), 100 steps min^−1^ (ΔEMR-100) and 120 steps min^−1^ (ΔEMR-120).

Factor	Spear-17	Ice Maiden
**Change in body composition**		
ΔBody weight (%)	− 6.5 (− 8.9; − 5.3)	− 7.8 (− 9.5; − 6.9)
ΔLean tissue weight (%)	1.0 (0.5; 1.5)	− 2.5 (− 3.6; − 1.7)*
ΔFat weight (%)	− 40.4 (− 43.7; -32.1)	− 30.4 (− 32.3; − 22.6)
**Change in energy expenditure during sleep and rest**		
ΔSMR-1/cal min^−1^ kg^−1^ lean tissue weight	0.2 (− 0.5; 1.0)	0.3 (− 0.7; 2.7)
ΔSMR-2/cal min^−1^ kg^−1^ lean tissue weight	0.0 (− 0.8; 0.3)	0.2 (− 0.6; 1.7)
ΔRMR/cal min^−1^ kg^−1^ lean tissue weight	− 0.4 (− 0.7; 0.1)	− 0.3 (− 0.8; 0.5)
**Change in energy expenditure during exercise**		
ΔEMR-80/cal min^−1^ kg^−1^ body weight	0.7 (− 2.4; 1.9)	3.3 (0.2; 6.3)
ΔEMR-100/cal min^−1^ kg^−1^ body weight	0.5 (0.0; 0.7)	0.7 (0.2; 1.2)
ΔEMR-120/cal min^−1^ kg^−1^ body weight	− 2.5 (− 3.2; 3.0)	1.1 (− 0.3; 2.5)

*Statistical significance between the data sets (*p* ≤ 0.05). It should be noted that the units of energy expenditure are cal min^−1^ kg^−1^ rather than the more usual kcal min^−1^ kg^−1^.

### Change in sleeping (ΔSMR1, ΔSMR2) and resting (ΔRMR) metabolic rate

**Figure 2:**
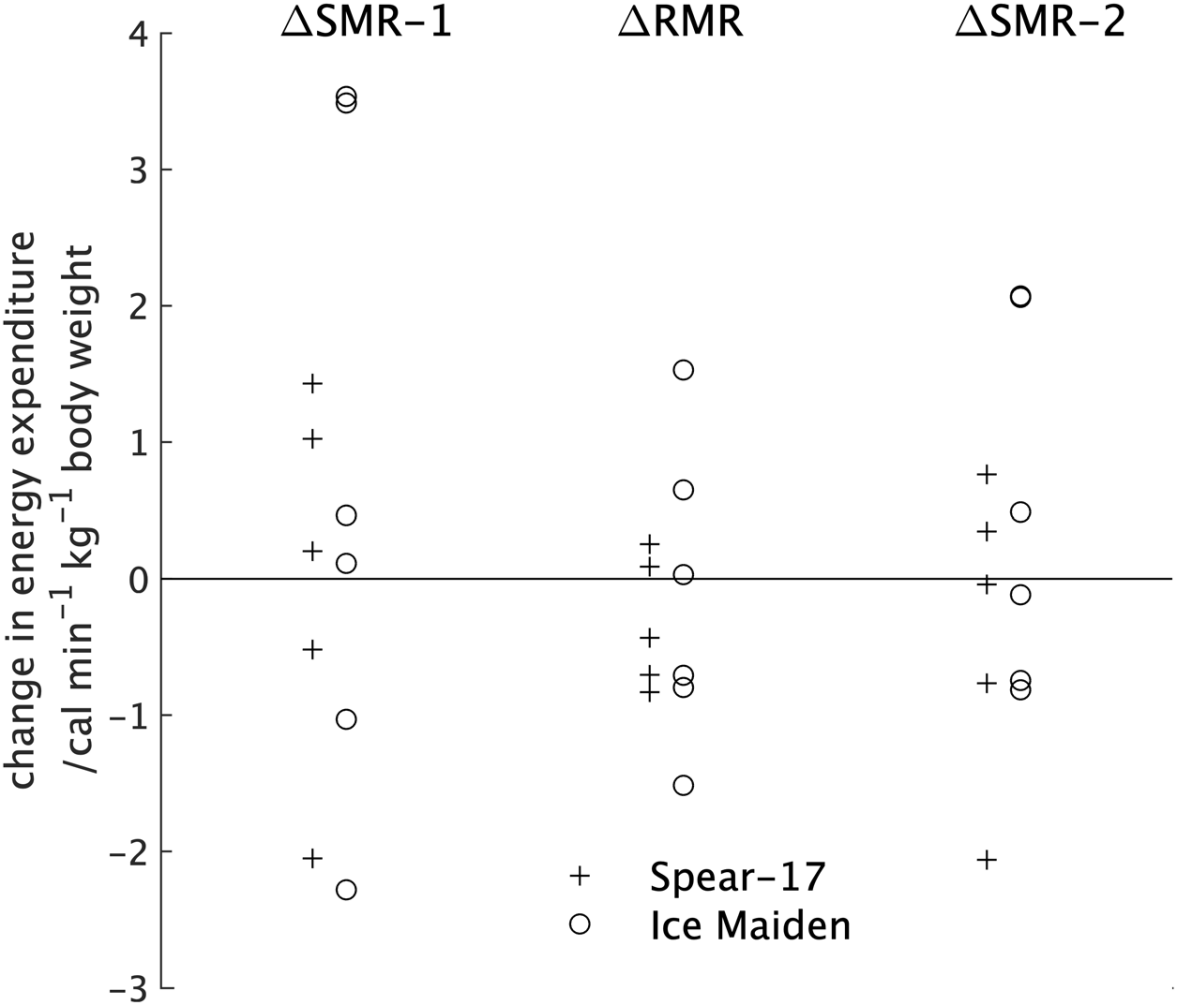
The percentage change in body composition between the pre- and post-expedition measurements for the individual participants in the two expeditions.

From the middle section of Table 1 it can be seen that the changes in energy expenditure during sleep and resting were small and not statistically significant, but that the range of values was large (Fig. 2). It should be noted that the values are given in units of cal min^−1^ kg^−1^ rather than the more usual kcal min^−1^ kg^−1^.

**Figure 3:**
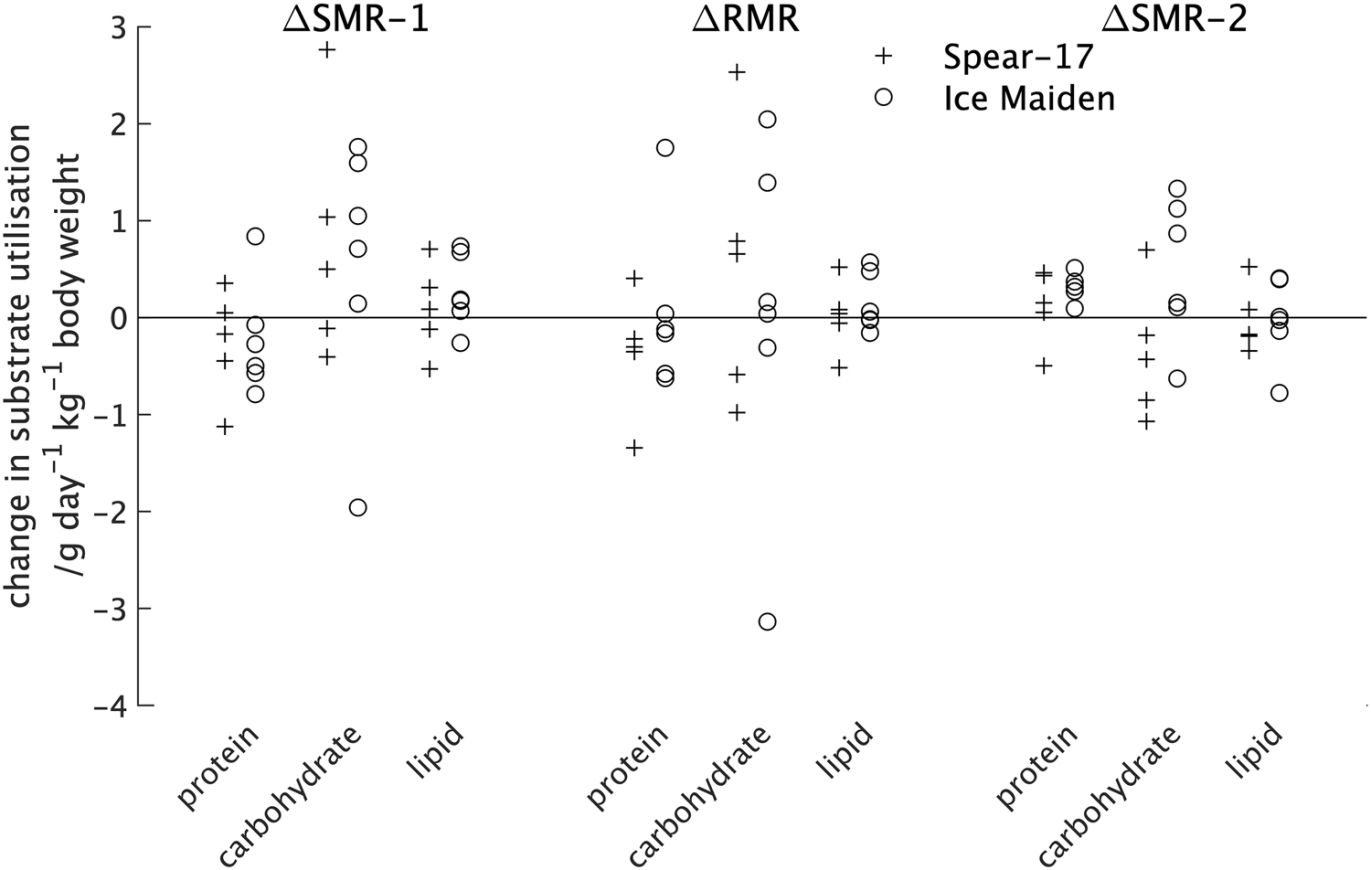
The change in substrate utilisation between the pre- and post-expedition measurements of the rest (ΔRMR) and two sleep periods (ΔSMR-1 and ΔSMR-2) for individual participants in the two expeditions.

The upper two sections of Table 2 shows the median and quartile (Q1; Q3) values for the difference in substrate utilisation for the two expeditions with individual values shown in Fig. 3. From these it can be seen there was little difference in the average value or spread of values for protein and lipid utilisation across the two sleep periods and the rest period or between the participants in the two expeditions. The modest change in carbohydrate utilisation between the pre- and post-expedition measurements for both expeditions was characterised all three measures (Table 2) but a much larger range of values than found for either the protein or lipid utilisations for all three measures (Fig. 3).

**Table 2:** The median, lower and upper quartiles (Q1; Q3) for the changes between pre- and post-expedition measurements of substrate utilisation during sleep (ΔSMR-1 and ΔSMR-2), rest (ΔRMR) and exercise (ΔEMR-80, ΔEMR-100 and ΔEMR-120).

Factor	Spear-17			Ice Maiden		
	Protein	Carbohydrate	Lipid	Protein	Carbohydrate	Lipid
**Sleep**						
ΔSMR-1/g day^−1^ kg^−1^ lean tissue	0.1 (− 0.1; 0.3)	0.5 (− 0.1; 1.0)	− 0.2 (− 0.4; 0.0)	0.2 (0.1; 0.6)	0.9 (0.3; 1.5)	− 0.4 (− 0.6; − 0.1)
ΔSMR-2/g day^−1^ kg^−1^ lean tissue	− 0.2 (− 0.2; 0.1)	− 0.4 (− 0.9; − 0.2)	0.2 (0.1; 0.4)	0.0 (− 0.1; 0.3)	0.5 (0.1; 1.1)	− 0.3 (− 0.3; − 0.1)
**Rest**						
ΔRMR/g day^−1^ kg^−1^ lean tissue	0.0 (− 0.1; 0.1)	0.7 (− 0.6; 0.8)	− 0.3 (− 0.3; − 0.2)	0.0 (0.0; 0.4)	0.1 (− 0.2; 1.1)	− 0.1 (− 0.5; 0.0)
**Exercise**						
ΔEMR-80/g day^−1^ kg body weight	0.1 (− 0.4; 0.1)	0.8 (0.8; 1.5)	− 0.5 (− 0.8; − 0.4)	− 0.1 (− 0.1; 0.1)	2.2 (1.0; 5.4)	− 0.4 (− 1.3; 0.8)
ΔEMR-100/g day^−1^ kg body weight	0.1 (− 0.4; 0.1)	1.6 (0.2; 3.2)	− 0.6 (− 1.0; 0.2)	− 0.1 (− 0.1; 0.1)	0.0 (− 1.0; 3.3)	0.2 (− 1.4; 0.6)
ΔEMR-120/g day^−1^ kg body weight	0.1 (− 0.4; 0.1)	2.5 (− 0.8; 3.5)	0.1 (− 1.6; 0.2)	− 0.1 (− 0.1; 0.1)	1.3 (0.3; 2.3)	− 0.4 (− 0.8; 0.1)

For the change in carbohydrate utilisation between the pre- and post-expedition measurements of sleep (ΔSMR-1 and ΔSMR-2) it should be noticed that: (a) whilst modest, the median value for participants in the Spear-17 expedition was positive for the first sleep period (ΔSMR-1) and negative for the second sleep period (ΔSMR-2) whilst that for participants in the Ice Maiden expedition was positive for both sleep periods; and (b) participants from both expeditions showed a reduction for the second sleep period (ΔSMR-2) when compared with the first sleep period (ΔSMR-1). A negative value indicates an absolute reduction in utilisation post-expedition when compared with the pre-expedition value. For the carbohydrate utilisation normalised to lean tissue weight there was no separation of the participants from the two expeditions (Fig. 3). When participants in the two expeditions were considered together with the median and quartile values (Q1; Q3) for the difference between the pre- and post-expedition measurements of carbohydrate utilisation during the two sleep periods were 0.7 g day^−1^ kg^−1^ of lean tissue (0.0; 1.3 g day^−1^ kg^−1^ of lean tissue) and 0.1 g day^−1^ kg^−1^ of lean tissue (− 0.5; 0.8 g day^−1^ kg^−1^ of lean tissue) for ΔSMR-1 and ΔSMR-2 respectively. Similarly, the difference in carbohydrate utilisation for all participants during rest (ΔRMR) was 0.2 g day^−1^ kg^−1^ lean tissue (− 0.4; 1.1 g day^−1^ kg^−1^ lean tissue). Thus, when participants from the two expeditions were considered together there was a larger carbohydrate utilisation in the post-expedition measurements for both sleep periods and during rest but the difference was largest for the first sleep period.

### Change in metabolic rate during exercise (ΔEMR-80, ΔEMR-100 and ΔEMR-120)

**Figure 4:**
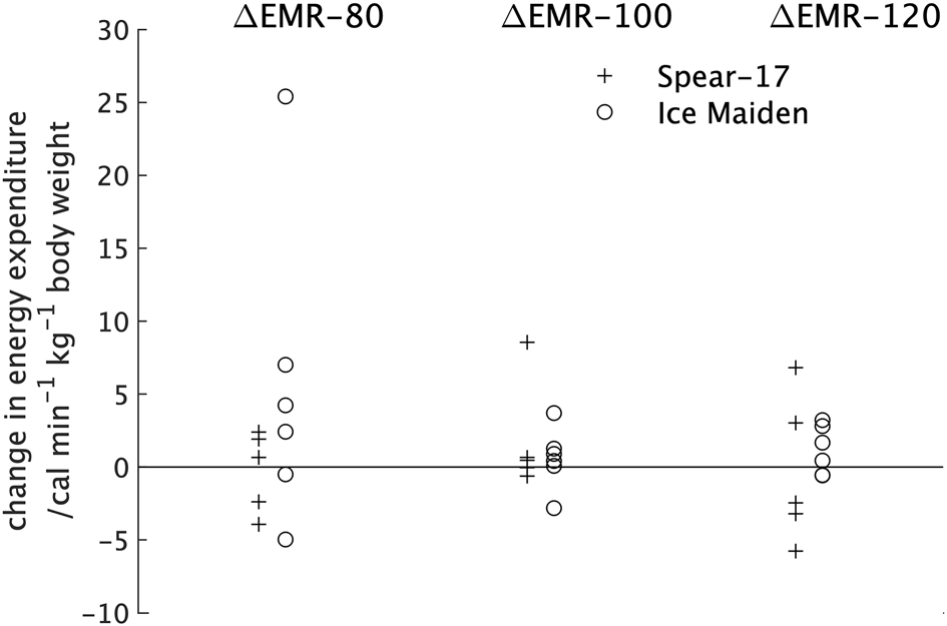
The percentage change in energy expenditure between the pre- and post-expedition measurements of the exercising metabolic rate at 3 intensities: 80 steps min^−1^ (ΔEMR-80); 100 steps min^−1^ (ΔEMR-100) and 120 steps min^−1^ (ΔEMR-120) for the individual participants in the two expeditions.

The median and quartile (Q1; Q3) values for the difference in energy expenditure between the pre- and post-expedition measurements during the three exercise intensities are given in the lower section of Table 1. As with the ΔSMR and ΔRMR values, the units are cal min^−1^ kg^−1^. For the 80 steps min^−1^ exercise, the average difference between the pre- and post-expedition measurements for the female participants was greater than that for the male participants but the difference was not statistically significant. The plot of the values for individual participants, Fig. 4, shows that one of the female participants had a much higher change in energy expenditure between the pre- and post-expedition measurements than the remaining participants. However, since non-parametric measures were used this single value will have minimal effect on the estimate of the average value or the determination of the U value for the Mann–Whitney test, there was no statistically significant difference.

**Figure 5:**
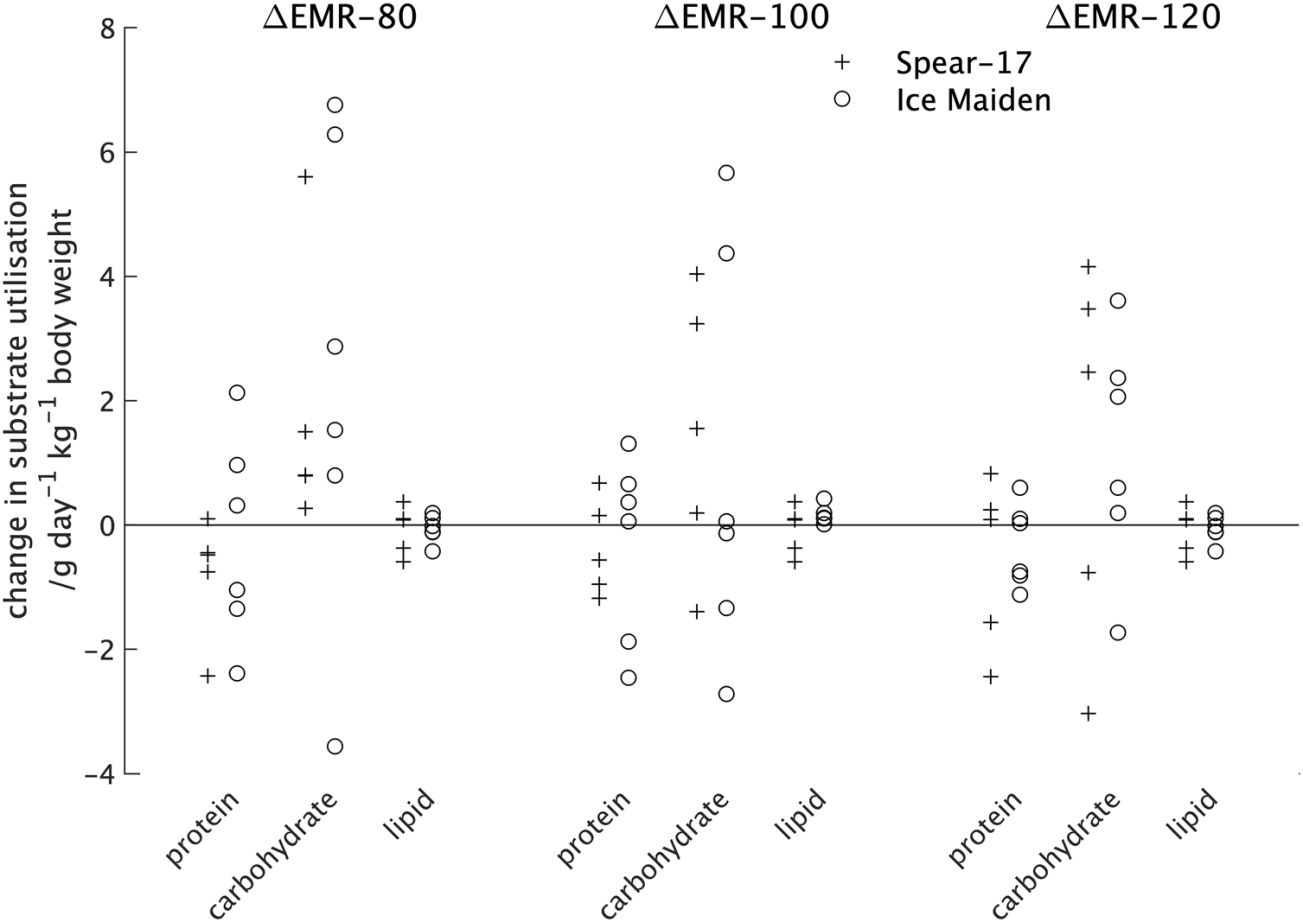
The change in substrate utilisation between the pre- and post-expedition during measurements of the exercising metabolic rate at three intensities: 80 steps min^−1^ (ΔEMR-80); 100 steps min^−1^ (ΔEMR-100); and 120 steps min^−1^ (ΔEMR-120) for individual participants in the two expeditions. Extreme values are shown at the edge of the plot together with their numerical values.

From Fig. 4 it can be seen that the range of value for the difference in energy expenditure between the pre- and post-expedition measurements for participants in the two expeditions is very similar if the single high value for the Ice Maiden participant at 80 steps min^−1^ is excluded. There was little difference in the values from the two expeditions at 100 steps min^−1^, but at 120 steps min^−1^ participants in the Spear-17 expedition had a non-statistically significant reduction in energy expenditure compared with participants in the Ice Maiden expedition. The lower part of Table 2 shows the average values for the difference in substrate utilisation between the pre- and post-expedition measurements from participants in the two expeditions. It should be noted that the difference in protein utilisation determined from 12-h urine samples, was the same for all exercise intensities, but values for each exercise intensity have been included in results tables and graphs for completeness. From Table 2 it can be seen that the pre- to post-expedition differences in protein and lipid utilisation were similar for the two expeditions but there was a larger difference in the carbohydrate utilisation. At 80 steps min^−1^ Ice Maiden participants had a higher difference in carbohydrate utilisation between the pre- and post-expedition measurements than the Spear-17 participants. However, this difference between expeditions was reversed for the 100 steps min^−1^ and 120 steps min^−1^ exercise intensities. None of the differences were statistically significant. A plot of the substrate utilisation for individual participants from the two expeditions for the different exercise intensities is (Fig. 5) shows a trend towards participants from both expeditions having a higher difference in carbohydrate utilisation between the pre- and post-expedition measurements at 80 steps min^−1^ that reduces as the exercise intensity increases.

This trend was not seen in the difference in lipid utilisation between the pre- and post-expedition measurements. There was a large overlap in the difference in carbohydrate utilisation between the pre- and post-expedition measurements for participants in the two expeditions for all exercise intensities. The distribution of these values for the 80 step min^−1^ intensity (ΔEMR-80, Fig. 5) was very similar to that for the first sleep period (ΔSMR-1, Fig. 3) and the resting metabolic rate (ΔRMR, Fig. 3). The median and quartile (Q1; Q3) values for the difference in carbohydrate utilisation between the pre- and post-expedition measurements for all participants studied for the three exercise intensities studied was 1.5 g day^−1^ kg^−1^ of lean tissue (0.8; 4.2 g day^−1^ kg^−1^ of lean tissue), 0.2 g day^−1^ kg^−1^ of lean tissue (− 0.7; 3.6 g day^−1^ kg^−1^ of lean tissue) and 2.1 g day^−1^ kg^−1^ of lean tissue (− 0.3; 3.0 g day^−1^ kg^−1^ of lean tissue) for ΔEMR-80, ΔEMR-100 and ΔEMR-120 respectively.

### Energy expenditure during the expedition

For both expeditions, the difference in the RMR measured before and after the expedition was small and not statistically significant^17,18^. Therefore the values for *BMR*_*temp*_ used in Eq. (1) were the average of the RMR values measured pre- and post-expedition. Summarised values for *BMR*_*temp*_ and the change in lean and fat weight for the two expeditions are shown in Table 3 together with the expedition data required for Eqs. (1) and (2).

**Table 3:** The expedition and measured values to allow the total energy and the energy due to activity during the expeditions to be determined from Eqs. (1) and (2).

	Spear-17	Ice Maiden
Expedition duration (days)	67	61
Nutritional energy available (E_nutr_) (kcal day^−1^)	6,500	5,000
Fraction of nutritional energy consumed (%)	92%	85%
Median (Q1; Q3) BMR_temp_ (kcal day^−1^)	2054 (2000; 2,106)	1793 (1629; 1865)
Median (Q1; Q3) change in fat weight (Δfat) (kg)	− 2.7 (− 8.3; − 2.4)	− 4.4 (− 5.3; − 3.5)
Median (Q1; Q3) change in lean weight (Δlean) (kg)	0.7 (0.4; 1.1)	− 1.4 (− 2.0; − 0.9)

**Figure 6:**
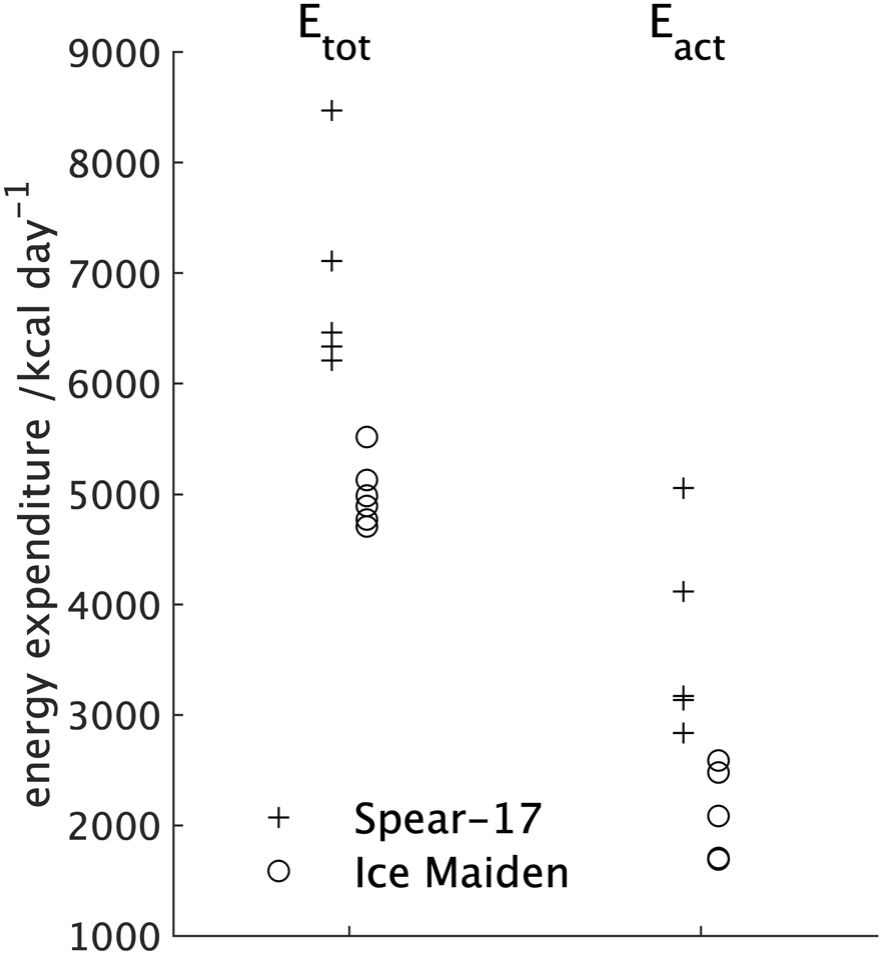
The total daily energy expenditure during the expedition (E_tot_) estimated using Eq. (2) and the energy expended on activity during the expedition (E_act_) estimated using Eq. (1) with *T*_*f*_ = 60% for individual participants in the Spear-17 and Ice Maiden expeditions.

The values of energy density used in calculating substrate utilisation from the O_2_/CO_2_ measurements in the whole body calorimeter were 9.461 kcal g^−1^ for fat, *ED*_*fat*_, and 4.316 kcal g^−1^ for protein, *ED*_*lean*_,^31^ Substituting theses values and the data from Table 3 into Eq. (2) gave median and quartile (Q1; Q3) values for the total energy expenditure during the expedition, E_tot_, of 6,461 kcal day^−1^ (6,335; 7,107 kcal day^−1^) for participants in the Spear-17 expedition and 4,939 kcal day^−1^ (4,803; 5,088 kcal day^−1^) for participants in the Ice Maiden expedition, a difference that was statistically significant (*p* = 0.004). The only value available for the time-averaged fractional increase in BMR for thermoregulation, *T*_*f*_, is the 60% measured by Stroud^15^. Substituting this together with the values for E_tot_ into Eq. (2) gave an energy expenditure on activity during the expedition, *E*_*act*_ of 3,174 kcal day^−1^ (3,136; 4,117 kcal day^−1^) for participants in the Spear-17 expedition and 2,282 kcal day^−1^ (1,801; 2,559 kcal day^−1^) for participants in the Ice Maiden expedition, a statistically significant difference (*p* = 0.004). The graph of total energy expenditure, *E*_*tot*_, and the energy expenditure on activity, *E*_*act*_, during the expedition for individual participants (Fig. 6) shows that values for participants in the Ice Maiden expedition were clustered together beneath a cluster of three of the five participants in the Spear-17 expedition. There were two outliers, both from the Spear-17 expedition, with values much greater than those from the other participants: one with values for *E*_*to*t_ and *E*_*act*_ of 8,470 and 7,107 kcal day^−1^, respectively and the other with values for *E*_*to*t_ and *E*_*act*_ of 5,054 and 4,117 kcal day^−1^, respectively.

**Figure 7:**
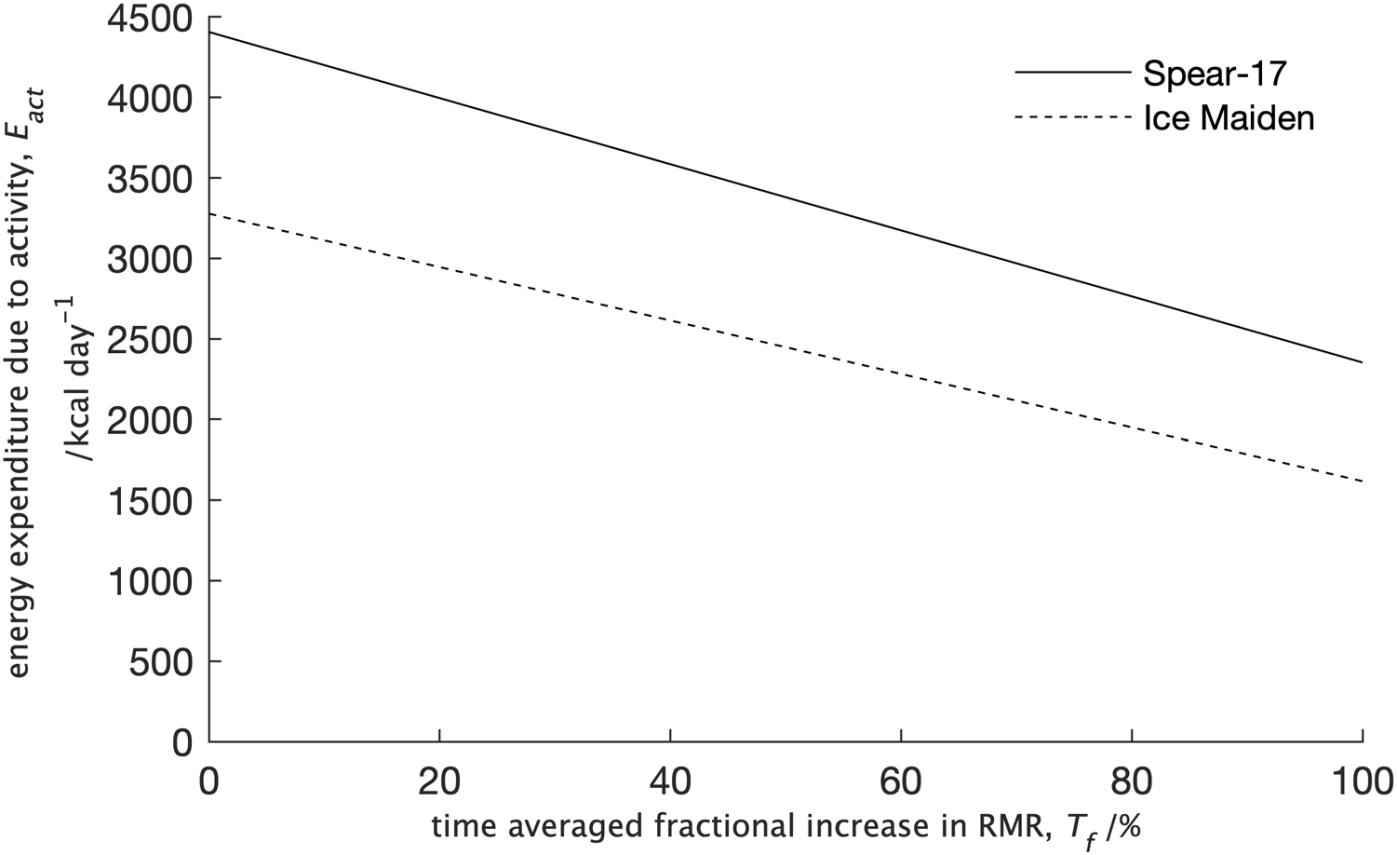
A plot of the median values for the energy expenditure on activity, *E*_*act*_, as the time-averaged fractional increase in RMR, *T*_*f*_, in Eq. (1) was varied from 0–100%.

The data in Fig. 6 were calculated using a value for *T*_*f*_ measured at rest^15^. In practice, this may not be a good estimate of the time-averaged value measured throughout the day. To investigate how the values of *T*_*f*_ affected the calculated energy expenditure due to activity the median values of this were plotted as *T*_*f*_ was varied from 0 to 100% (Fig. 7).

As predicted by Eq. (1), Fig. 7 shows the energy expenditure due to activity decreases as the time-averaged percentage increase in RMR, *T*_*f*_, increases. From the figure, it can also be seen that the difference in the median energy expenditure between the two expeditions decreases as *T*_*f*_ increases.

## Discussion

We have previously reported a detailed analysis of the energy expenditure and substrate utilisation from participants in the Ice Maiden^18^ and Spear-17^17^ expeditions. Whilst these expeditions were of similar duration and length, comparing energy expenditure and substrate utilisation across the two expeditions is challenging because differences in body size and composition will affect the measurements. A common approach is to normalise rest and sleep data to lean weight on the assumption that fat is metabolically inactive^35–37^ However, there are metabolic processes involving fat that are dependent on the amount present^35,36^. A more robust normalising factor is undoubtedly obtained if this metabolising fat contribution is accounted for, but to do that in a study population requires covariance^35^ or correlation^36^ techniques. In this study we would need to do this for Spear-17 and Ice Maiden participants independently both before and after their expeditions to avoid discontinuities; the number of participants (5 and 6 respectively) is not sufficient to give robust results. As a result of this together with participants not being obese, we have followed the recommendations of Arch et al.^35^, if in the absence of more robust methods, as a minimum, normalised non-exercise measures to lean weight. The stepping exercises involve a cyclical change in potential energy determined by body weight and therefore the energy expenditure and substrate utilisation values have been normalised to body weight. Other metabolic measures traditionally cited include diet induced thermogenesis (DIT) and Total Energy Expenditure (TEE) during the 24-h measurement period. During much of the daytime component of the 24-h measurement period there is undocumented very low levels of physical activity for which the energy expenditure will be an unknown function of both lean tissue weight and body weight. Therefore the measure has not been included in the analysis reported in this paper. Similarly, DIT has been omitted from the current analysis as it is not directly measured but rather was determined using the intercept method^33,38^ where the effect of data normalisation is unknown.

The modest percentage change in body weight suggests that the composition and quantity of the diet was appropriate to both the environment and the physical effort required to pull the sledges. The available diet for Spear-17 participants delivered 6,500 kcal day^−1^ whilst that for Ice Maiden participants delivered 5,000 kcal day^−1^. Importantly, even though not all the available calories were consumed, there was minimal loss of lean tissue weight from participants in the Ice Maiden expedition whilst participants in the Spear-17 expedition gained a small amount of lean tissue weight; a difference that gave the only statistically significant difference between the two expeditions in the factors studied. Taken together the results for the change in body composition for both expeditions suggests that whilst there was negative Energy Availability (EA), this was modest.

The median of the total energy expenditure during the expedition for participants in the Spear-17 expedition (6,461 kcal day^−1^) was consistent with an activity estimate of 6,100 kcal day^−1^^14^. Noting the two outliers, with energy expenditure of 7,107 and 8,470 kcal day^−1^, the total energy expenditure by participants in the Spear-17 expedition was also consistent with the average of 7,390 kcal day^−1^ measured using doubly labelled water (DLW) on two male subjects during polar expeditions^10,12^. It is interesting to note that in both Arctic^12^ and Antarctic^10^ expeditions, the older of the two participants had a substantially higher daily energy expenditure than the younger. The second highest value from participants in the Spear-17 expedition was for the oldest member who had the smallest lean tissue weight from the body composition measures and, as a result, the smallest RMR values and hence the smallest *BMR*_*temp*_ value. There is anecdotal evidence from the expedition team that the Spear-17 participant with the highest value for total energy expenditure spent periods of the expedition walking rather than skiing due to a technical issue with a ski; walking in such conditions would require much greater energy expenditure. The median total energy expenditure for participants in the Ice Maiden expedition, 4,939 kcal day^−1^, was much lower than that for participants in the Spear-17 expedition and inconsistent with both activity based estimates from previously measured values^10,12,14^. Despite being statistically significant, the difference in the median energy due to activity, *E*_*act*_, between the two expeditions was surprisingly small (892 kcal day^−1^) given two key differences between the expeditions that would suggest much greater energy expenditure from participants in the Spear-17 expedition. Firstly, due to the single versus dual resupply strategy, the sledges on the Spear-17 expedition weighed a maximum of 120 kg; whilst those on the Ice Maiden expedition weighed a maximum of 80 kg. Secondly, whilst both expeditions crossed un-traversed ice, the initial section of the Ice Maiden expedition (Leverett Glacier to the South-pole) used a semi-prepared supply track. To put the difference in energy expenditure due to activity during the expeditions into perspective, it is 43% and 50% of the RMR measured in a temperate climate for the Spear-17 and Ice Maiden participants respectively (Table 3). The values of *E*_*tot*_ for participants in the all-male Spear-17 expedition were consistent with measurements made on male participants in previous expeditions whereas those for participants in the all-female Ice Maiden expedition were not. This suggests the calculated energy expenditure on activity, *E*_*act*_, by participants in the Ice Maiden expedition is too high. Whilst the equation for determining the total daily energy expenditure, *E*_*tot*_, from Eq. (2) must be treated with some caution because of the assumptions inherent in it, we can postulate that the too high value for the energy due to activity, *E*_*act*_, is the result of a too low value for the energy expenditure on thermoregulation through non-shivering thermogenesis (*T*_*f*_ × *BMR*_*temp*_) in Eq. (1). Since *BMR*_*temp*_ was taken from measured values of the RMR, it follows that the estimate for the time-averaged fractional increase in BMR due to the low temperatures, *T*_*f*_, of 60% for the Ice Maiden expedition was too low. The energy for thermoregulation is higher in men when compared with women as a result of the greater lean tissue weight and body surface area^39^; but a larger value of *T*_*f*_ for the Spear-17 participants would tend to reduce the difference in the energy available for activities between the two expeditions, rather than increase it (Fig. 7). In addition, we have previously shown through comparisons with other studies that a value of 60% for *T*_*f*_ is consistent with the energy for thermoregulation for participants in the Spear-17 expedition. Both expeditions were equipped with clothing appropriate to the Antarctic environment leaving only the face exposed. Experimentally, facial cooling has been shown to cause a less than 5% increase in the metabolic rate in men when exercising^14^ so this was unlikely to explain the increase in the energy for thermoregulation in the Ice Maiden participants. A possible explanation for the higher energy for thermoregulation by participants in the Ice Maiden expedition stems from the greater effort required by participants in the Spear-17 expedition to travel over previously un-traversed ice. The higher muscle activity of the Spear-17 participants would increase the energy due to activity, *E*_*act*_, whilst also increasing the heat generated due to the inefficiency of muscle activity resulting in reduced energy for non-shivering thermogenesis and hence a smaller value for *T*_*f*_ in Eq. (1) compared with the value for participants in the Ice Maiden expedition.

The value for *T*_*f*_ is a time-averaged value will depend on the intensity and duration of physical activity. Without additional measurements made during the expedition it is impossible to determine what the numerical value for the Ice Maiden expedition should be, only that it is greater than 60%. The current analysis has used a single value of *T*_*f*_ for all participants in an expedition. However, the wide variation in energy expenditure, particularly amongst participants in the Spear-17 expedition, suggests that values for individual participants may be more appropriate.

It is recognised that measurements to establish personalised values is challenging, made more challenging under expedition conditions. Isotope techniques, which have been used on previous expeditions^11,12^, could have given a measurement of the total daily energy expenditure, *E*_*tot*_, replacing the estimate obtained from Eq. (2) but approvals and logistics constraints prevented this. A non-laboratory study on thermoregulation where participants undertook strenuous work in hot and cold environments^7^ used a modelling and qualitative scoring system to determine the energy required for thermoregulation^40^. This system, which was designed for short periods of well-defined activity, could potentially be adapted for use on polar expeditionary journeys but the time required by participants for measurement and record-keeping to obtain robust values may be prohibitive. A more realistic approach to determining the energy expenditure on activity and thermoregulation during expeditions may be the next generation of body-worn biosensors^41^.

The differences between the pre- and post-expedition energy expenditure during sleeping, resting and exercise for the participants in the two expeditions were small. There was no statistically significant difference in these measures between participants from the two expeditions. If energy expenditure is considered a proxy for metabolic activity then these results suggest that there was little metabolic consequence to the participants for either of the expeditions studied. Importantly, there was no difference in the metabolic consequence of men and women undertaking expeditionary travel when appropriately trained and with an appropriate diet. The participant from Spear-17 who left the expedition at the South Pole did so because of extreme exhaustion.

The changes in protein and lipid utilisation between the pre- and post-expedition measurements were small for participants from both expeditions and there was no statistically significant difference between participants in the two expeditions. The range of values for the change in carbohydrate utilisation between the pre- and post-expedition measurements was much higher than the other substrates for participants from both expeditions, but once again there was no statistically significant difference between participants in the two expeditions. The first sleep period occurs before the 24-h measurement period and measurements may be affected by activities and diet prior to arrival for the start of the study. Within the 24-h measurement period, the upper quartile measured across participants in both studies for the pre- to post-expedition change in carbohydrate utilisation was higher during resting (ΔRMR) when compared with the second sleeping period (ΔSMR-2) and higher during the lowest intensity exercise (ΔEMR-80) when compared with higher intensity exercise (ΔEMR-100 and ΔEMR-120). The measurements used to determine the values for ΔRMR and ΔEMR-80 were both performed when participants were fasted whilst those for ΔEMR-100 and ΔEMR-120 are performed when participants were fed. We have previously reported that some, but not all participants in the Spear-17 expedition had increased carbohydrate utilisation when fasted which decreased when fed in the post-expedition measurements, a decrease that became absolute during sleep, rest and very low levels of physical activity^17^. These changes were not seen in the female participants in the Ice Maiden expedition^18^. However, the number of participants in each study was small and the spread of values large with a large overlap of values between participants in the two expeditions. In a study where lightly clad men and women were exposed to air temperatures at 5 °C, a higher carbohydrate utilisation was found in men when compared to women, but the spread of values for individual participants was large^39^. The analysis reported in this paper show that the difference between pre- and post-expedition carbohydrate utilisation is larger in a subset of participants when they have fasted; a difference which reduces when they are fed. This subset includes participants from both expeditions. However, the impact of the environmental and physical activity on diurnal substrate utilisation, particularly in the fasted state, should be the focus of further investigation.

The same energy density values used in the determination of substrate utilisation from the measured O_2_ and CO_2_ in the whole body calorimeter were used for the determination of energy expenditure during the expeditions from Eqs. (1) and (2). Livesey and Elia^42^ showed that the chemical composition of substrates affects their energy density and the authors compare values for the energy density of fat and protein obtained from different studies. Using these data, the variation in energy density for fat across the different studies was about 2% and for protein was much higher at about 14%. A simple sensitivity analysis on our data showed that a ± 2.5% change in the energy density value of fat produced a maximum change in the calculated energy values during the expedition of < 40 kcal day^−1^ (< 1.2%) and a ± 15% change in the median energy density of protein produced a maximum change < 125 kcal day^−1^ (< 2%) in the median calculated energy values.

Perhaps the most important finding from this current work is that no difference was found between participants in the two expeditions for any of the energetics measures studies and therefore between male and female participants undertaking sustained expeditionary polar travel. The large range of values and the extensive overlap in values from participants in the two expeditions suggest that the differences have their origins in individual adaptation to an extreme environment rather than a systematic difference between men and women. This is consistent with findings at altitude where some studies have reported increased carbohydrate utilisation in men^4^ whilst other studies have failed to find an increase in men^5^ or women^6^.

Previous research on healthy volunteers undertaking activities in extreme environments has generated new medical and physiological knowledge, including insights into the human skeleton, energetics^43,44^, cardiac function^45^, cerebral blood flow^46^, treatment of lung disease^21^ and the pre-hospital treatment of emergencies^47^. The work reported in this paper demonstrates the importance of appropriate physical and nutritional preparation before and appropriate nutrition during physical and physiological challenges. In a healthcare context, this includes the planning and post treatment care of patients undergoing surgery, and the planning intra- and post-treatment care of patients undergoing radiotherapy and chemotherapy. This is an area for research that we believe has received inadequate attention in the past.
